# Multi-color electron microscopy by element-guided identification of cells, organelles and molecules

**DOI:** 10.1038/srep45970

**Published:** 2017-04-07

**Authors:** Marijke Scotuzzi, Jeroen Kuipers, Dasha I. Wensveen, Pascal de Boer, Kees (C.) W. Hagen, Jacob P. Hoogenboom, Ben N. G. Giepmans

**Affiliations:** 1 Dept. Imaging Physics, Delft University of Technology, Delft, The Netherlands; 2 Dept. Cell Biology, University Medical Centre Groningen, University of Groningen, Groningen, The Netherlands

## Abstract

Cellular complexity is unraveled at nanometer resolution using electron microscopy (EM), but interpretation of macromolecular functionality is hampered by the difficulty in interpreting grey-scale images and the unidentified molecular content. We perform large-scale EM on mammalian tissue complemented with energy-dispersive X-ray analysis (EDX) to allow EM-data analysis based on elemental composition. Endogenous elements, labels (gold and cadmium-based nanoparticles) as well as stains are analyzed at ultrastructural resolution. This provides a wide palette of colors to paint the traditional grey-scale EM images for composition-based interpretation. Our proof-of-principle application of EM-EDX reveals that endocrine and exocrine vesicles exist in single cells in Islets of Langerhans. This highlights how elemental mapping reveals unbiased biomedical relevant information. Broad application of EM-EDX will further allow experimental analysis on large-scale tissue using endogenous elements, multiple stains, and multiple markers and thus brings nanometer-scale ‘color-EM’ as a promising tool to unravel molecular (de)regulation in biomedicine.

Precise identification and ultrastructural localization of molecules, organelles, cells and other biological structures is a key step to unravel how these act to regulate biology. Electron microscopy (EM) provides nanometer-resolution images of the cellular ultrastructure, which can be automatically collected to allow large field-of-view or three-dimensional imaging at high magnification^1^. However, data analysis is hampered by visual interpretation of grey-scale images, especially for finding rare or unanticipated events in large datasets. Fluorescence microscopy aids to identify biomolecules^2^,^3^,^4^,^5^, but lacks structural context. Correlated light microscopy and EM (CLEM)^6^,^7^ allows fluorescence-guided analysis of EM data, but fluorescence retention during EM sample preparation and overlay of images differing order-of-magnitude in resolution may be technically challenging^6^,^7^. In search for a broadly implementable technique to define molecules, organelles and cells at high resolution within mammalian tissue, we decided to implement element-guided identification using energy dispersive X-ray analysis (EDX). In mammalian tissue, detection sensitivity of typically low percent elements in combination with high count rates from carbon and oxygen as well as radiation damage have limited broad application of EDX imaging for a long time. EDX spectroscopy and imaging on cryo-fixed tissue has been pioneered by Somlyo and coworkers^8^,^9^,^10^, and pioneering studies have mainly focused on detection of a few selected elements in small regions at relatively low resolution (see for example^11^,^12^). Leapman and co-workers applied and pioneered electron energy loss spectroscopy (EELS) in transmission EM to discriminate cells based on sequential analysis of three elements^13^, and recently Tsien and coworkers presented EELS-based two-color discrimination of localized deposits of lanthanides^14^. EDX allows direct identification of many elements in parallel, either present endogenously and/or introduced by staining or labeling, at high count rates using the latest generation of silicon drift detectors (SDD). We find that this paves the way for straight-forward high-resolution elemental mapping in mammalian tissue compatible with standard EM protocols. The resulting elemental color-maps can be overlaid with the conventional EM data to allow data-mining based on composition *and* structure, rather than morphology only. We apply this approach in large field-of-view EM (“nanotomy”)^15^,^16^,^17^ on pancreas from a rat model for Type 1 diabetes (T1D). EDX not only allows us to identify organelles and biomolecular labels at high resolution, but also to show that distinct granules have typical elemental fingerprints. EDX-guided elemental fingerprinting in combination with large-scale EM reveals cells that contain both hormones and exocrine granules in the pancreas. Given that a sensitive EDX SDD is a standard retrofit add-on to electron microscopes, we foresee broad application of such a technique in both label free and studies using exogenous tracers. This approach is applicable to both life science and biomedical research. Such a technique increases the depth of information with the color coding of structures based on their elemental profile and brings an objective analysis tool to EM imaging.

## Results

**Figure 1:**
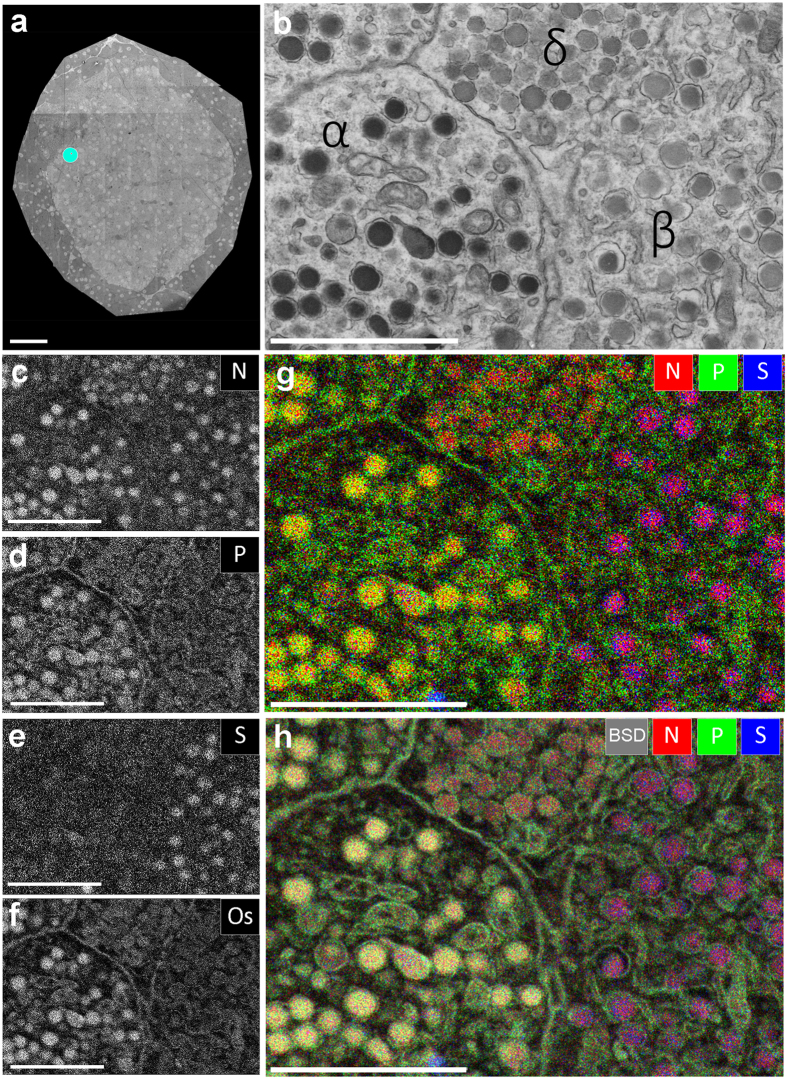
EDX defines cell-types and subcellular structures and organelles in EM images. (**a**) 100 nm thin cross-section of an entire rat islet of Langerhans imaged at 2.5 nm pixel size (STEM). Full high-resolution data is available via nanotomy.org (see suppl. material). (**b**) Area of interest (indicated in a; cyan dot) shows parts of four cells with different granules based on grey levels and morphology. (**c**–**f**) Elemental content in the ROI as indicated. (**g**) Overlay of N (red), P (green) and S (blue) allows identification of cells and granules based on elemental content. (**h**) Overlay of back scatter ICD image (greyscale) over the color-image of g. Bars: 50 μm (**a**) and 2 μm (**b**–**h**). Maps of other elementals are available as Fig.S1.

Large-scale EM of standard prepared rat pancreas fixed with aldehydes and osmium, embedded in epon (Fig. 1a; full resolution at www.nanotomy.org) was recorded using scanning transmission EM^17^,^18^. An endocrine area with three different cell types was selected (Fig. 1b). Traditional visual grey-scale analysis presumptively identifies these as a somatostatin-producing delta cell (top middle), a glucagon-producing alpha cell (left) and an insulin-producing beta cell (right). EDX analysis reveals informative maps of nitrogen, phosphor, sulphur and osmium (N, P, S, Os respectively) localization (Fig. 1c–f; and Fig. 1g,h for overlays; see Fig. S1 for more elemental results). N is abundant in all granules, as expected for highly concentrated peptides, irrespective of presumed cell identity. S is most abundant in insulin granules, as expected from the high cysteine content. The glucagon granules are found to stand out in the P map, whereas somatostatin shows neither pronounced S or P and is thus recognized on the sole presence of N. These compositional differences between granules are also revealed in qualitative comparison of the full EDX spectra (Fig. S4) and are furthermore reproduced using alternative, osmium-free sample preparation (Fig. S5). P maps also show condensed heterochromatin in the nucleus and the very dense endoplasmic reticulum network of the exocrine cells where P-rich RNA is translated. At high resolution also mitochondria are in the P map, which may reflect the abundance of phospholipids and ATP production. Overlay of the N, P, and S maps (Fig. 1g) clearly discriminates, in color indicating their elemental fingerprint, the separate granules. Not surprisingly, addition of the Os map, used as fixative and the only EM contrasting agent, adds the electron density determined by back scattered electron detection (BSD) from the EM image (Fig. 1h).

**Figure 2:**
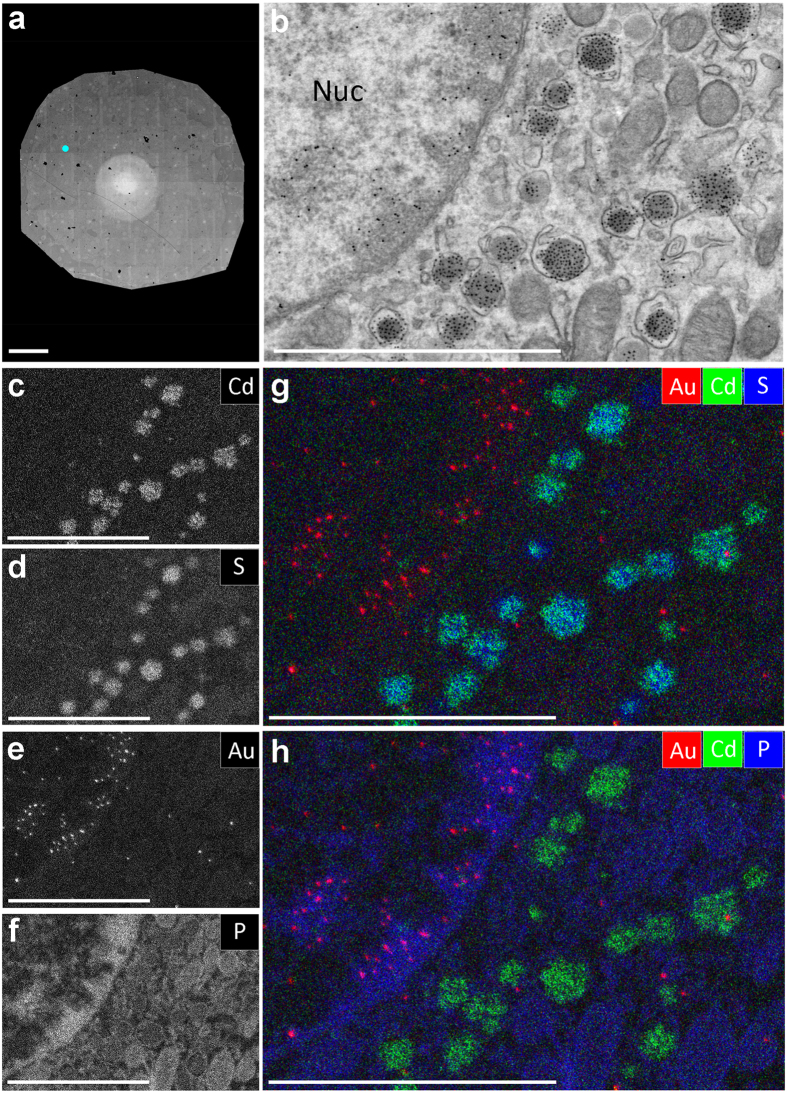
Immuno-based identification of peptides and G4-DNA structures and endogenous elements. (**a**) Section of an islet immuno-labeled for G4 (10 nm immunogold) and insulin (QD655). The white area in the middle is an electron-beam pre-exposure artefact, but not hampering data analysis. Full resolution is available via nanotomy.org. (**b**) Zooming into the data reveals gold particles in the nucleus (upper left part) and quantum dots in insulin granules. Labels are identified based on grey levels and morphology. (**c**–**f**) Elemental content in the ROI as indicated. (**g**,**h**) Overlay of Au (red), Cd (green) and S (blue; g) or P (blue; h) allows identification of G4 structures based on gold presence and insulin granules based on Cd content. Note the localization of Cd on S-enriched insulin granules (**g**) and localization of Au to heterochromatin regions enriched in P in the nucleus (h). Bars: 50 μm (a) and 2 μm (**b**–**h**). See for more elements Fig. S2 and spectral analysis Fig. S6.

High-resolution definition of targets by EDX can further aid in molecular identification or assist in validation when applied to the recognition of elements specifically deposited at biomolecules of interest using antibody labeling. We choose a 1H6 monoclonal antibody that was raised against guanine quadruplexes (G4)^19^ and an anti-insulin antibody^18^, for which we previously established immunolabeling on epon sections^18^. Primary antibodies were subsequently labeled with secondary antibodies conjugated to gold (Au) and Cadmium-Selenide (CdSe)-based quantum dots (QDs), elements that are (nearly) absent in mammals^20^. Nanotomy was performed (Fig. 2a; www.nanotomy.org) and an area was selected for EDX analysis (Fig. 2b). Shown is part of an insulin-producing beta cell, with in the top left a nucleus with euchromatin (white, light grey) and darker heterochromatin, especially present near the nuclear envelope. The 1H6 antibody developed against G4 structures shows strong reactivity in areas of heterochromatin^19^, as also can be deducted from the electron-dense gold particles. In the cytosol, the presence of mitochondria and insulin-granules is clearly seen, the latter decorated with the electron-dense quantum dots (Fig. 2b). EDX analysis for Cd-based QDs, S-enriched insulin, Au (G4) and P-rich heterochromatin (Fig. 2c–f respectively; Fig. S4 for full elemental results) reveals that both Au and QDs are readily detected by EDX. Thus unambiguous identification of targets in biosamples by elemental analysis can be performed in addition to analysis by grey levels, size, or shape^21^,^22^. Note the high signal to noise ratio and co-localization of Cd (QDs; green) with S (insulin; blue), but not Au (1H6 antibody raised against G4; red) in the overlay (Fig. 2g). Similarly, Au (G4, red) localizes to P-rich areas in the nucleus (heterochromatin, blue; see also spectral data in Fig. S5), whereas the Cd (QDs, green) signal is enclosed within P rings (blue) that likely represent phospholipid membranes of the vesicles (Fig. 2h). Thus, EDX analysis allows for high resolution identification of targets in conjunction to endogenous elemental composition in mammalian tissues.

**Figure 3:**
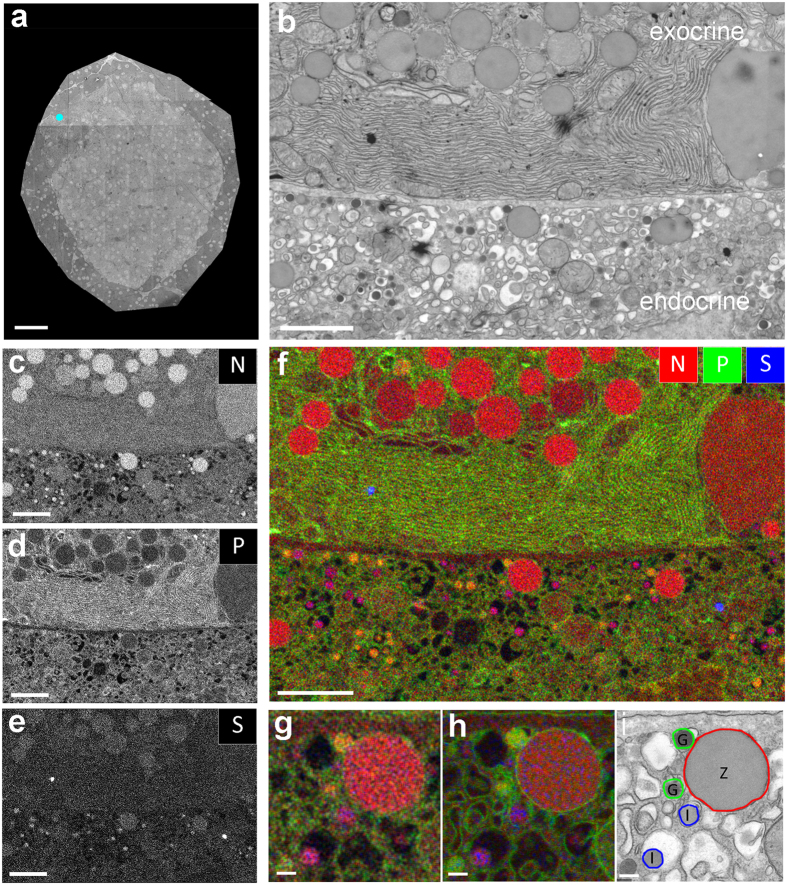
EDX analysis reveals single cells that contain exocrine zymogen granules with endocrine glucagon and insulin. (**a**) Overview as in Fig. 1a. (**b**) Area indicated in (a) by the cyan dot with parts of two cells with different granules based on grey levels and morphology. (**c**–**e**) Elemental maps as indicated. (**f**) Overlay of N (red), P (green) and S (blue) allows identification of cells and granules. (**g**) Zoom of the center part of f, note the small purple and yellow granules, as well as the big red granule, all present in the same cell. (**h**) Same region, with P and S combined with the backscatter image, which was reverted to red. Note the membranes around the vesicles. (**i**) Interpretation of the EDX data on the large scale STEM image showing the presence of three distinct granules in an endocrine cell. Bars: 50 μm (a), 2 μm (**b**–**f**) and 0.2 μm (**g**–**i**). See for more elements Fig.S3.

**Figure 4:**
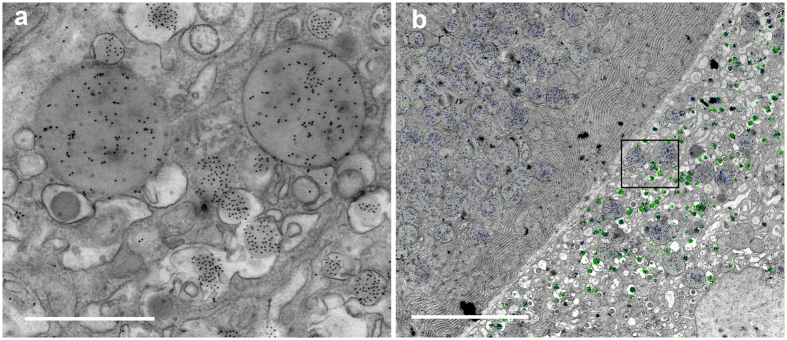
Distinct granules in the same cell determined by immuno-labeling. (**a**) Double immuno-labeling of amylase (zymogen granules) with 10 nm immunogold and insulin with QD655 shows the appearance of distinct granules in the same cell based on morphology and electron density of the nanoparticles; immunogold is rounder and darker compared to the more rectangular and less electron dense QDs. Note that also unlabeled glucagon granules are present in the same cell. (**b**) Semi-automated nanoparticle annotation using the Fiji macro “golddigger” shows that distinct granules in the same cell occurs specifically in the islet (right) with the co-appearance of amylase (nanogold, blue) and insulin (QD655, green) labeling, and solely amylase (blue) labeling in the exocrine pancreas (left). Bars: 1 μm (**a**) and 5 μm (**b**).

To explore EDX for biomedical microscopy, we used embedded material from our previous studies^15^, namely a diabetic prone rat. 90% of these rats spontaneously develop diabetes, with the blood sugar level as an indication for the diabetic state. This experimental animal had not developed diabetes, and showed no signs of insulitis^15^ (Figs 1a, 2a, 3a and dataset). Unexpectedly, typical distinct vesicles within one cell are present at multiple locations. These multi-vesicle containing cells are at the border of the endocrine Islet of Langerhans and the surrounding exocrine tissue, the latter readily identifiable by the abundant endoplasmic reticulum and large zymogen vesicles (Fig. 3b, top). The adjacent cell has all characteristics of an endocrine cell (Fig. 3b, bottom). Note the presence of the small granules with a halo (typical for insulin) and other small granules showing an overall high electron density (typical for glucagon), but also large vesicles that resemble the zymogen granules in the exocrine tissue. Using the elemental characteristics to discriminate granules (Fig. 1), we analyzed the distribution of N, P and S (Fig. 3c–e; full elemental results in Fig. S3). Three distinct granules with characteristics of zymogen (N; red), glucagon (N in red and P in green, yellow appearance of coincidence) and insulin (N in red and S in blue, purple appearance) are present (Fig. 3f), which is more prominent at higher zoom (Fig. 3g). Color-coding of the backscatter image in green, a signal mainly caused by Os, allows to superpose the signal created by Os enriched in membranes (Fig. 3h). Interpretation of the multi-hormone and zymogen containing area is modelled on the STEM image (Fig. 3i). Thus, only with EM three distinct granules can be conclusively identified within the same cell and EDX shows the different composition based on different elemental ratios, thus without prior knowledge or anticipated labeling. Based on these new observations, we decided to substantiate our notion probing protein content, and subsequent optimization of double-immunolabeling confirmed our findings (Fig. 4).

## Discussion

EDX imaging strongly aids in analysis of EM-data of mammalian tissue. Compared to previous approaches using EDX-imaging^11^,^12^, which to our knowledge, have mostly been done on cryo-samples, freeze dried samples and/or unstained epon^23^,^24^ we used chemical fixation, staining, and dehydration. Nanotomy allows analysis of complete cross sections of Islets of Langerhans, although the use of labels to detect proteins in an immunobased manner may benefit from Tokuyasu-sample preparation, which will be better compatible with epitope recognition, but this is typically performed on smaller sections (reviewed in refs 6,7). Together with EM-based enhancements (silver) and immuno-labeling nanoparticles this enables recognition of multiple targets at the EM resolution level.

The use of high-sensitivity silicon drift detectors (SDDs)^25^ with high-current, high-resolution SEMs now allows determination of variations in elemental composition and elemental fingerprinting from the subcellular to the nanoscale level. These may be due to natural or enriched variations of endogenous elements, introduced stains (like osmium), and/or specific labels as shown for immunotargeted gold and QDs. Elemental detection has been explored before, mostly EELS focusing on quantitative determination of concentrations of typically one or two elements at the (sub)cellular level (reviewed in refs 26,27). The Leapman lab pioneered EELS, which is capable of discriminating insulin and glucagon in cells^13^. An early study on the use of QDs^21^ for immunolabeling directly noted the possibilities of elemental detection (EELS), posing that the diversity may be increased by using other elements in nanoparticles^21^.

Implementation of EDX to discriminate ultrastructural features in the rat model for Type 1 diabetes (T1D) revealed distinct granules, believed to be cell-specific, within the same cells (Fig. 3), which have been substantiated by immunolabeling (Fig. 4). Such diversity has been reported in artificial differentiation protocols^28^,^29^,^30^. T1D is caused by loss of the insulin-producing beta cells due to an auto-immune reaction of which the trigger is not known. More recently, the exocrine pancreas has been proposed to play a role in the destruction of beta cells, either by enhanced infiltration of immune cells in an animal model^31^ or by the notion that T1D patients have a 30% reduction in pancreas weight^32^. Our observation may also hint to a malfunctioning of differentiation, or may reflect an exocrine/endocrine cell interaction, which opens the intriguing possibility that exocrine cells may interfere with beta cells. Thus the existence of mixed cells is readily identified by EDX. Although we only observed this in Islets of two diabetic rats (total of 32 cells), not in two controls, a conclusive statement whether or not this phenomenon relates to a (pre)diabetic state should await results from a follow-up study. This will include examining human pancreas^32^, but is beyond the scope of this paper.

EDX has been used to discriminate healthy or diseased cells based on elemental expression, or to address whether certain elements are enriched^33^. Recently in an isolated cell model the potential to use elemental analysis and subcellular features was shown. Combination of EDX with EELS to explore additional elements with weak X-ray signals may thus further increase the elemental palette^8^. EDX can be implemented in an existing TEM or SEM, is compatible with epon-embedded material, straightforward, and compared to EELS analysis, does not need a priori selection of imaging windows. Another mode of color-EM, CLEM, is widely used to localize targets typically between 0.05–1 μm^2^,^3^,^4^,^5^. We foresee an integrated^34^ approach, where EDX and CLEM not only complement, but also allow crossing scales from rapid fluorescence screening of large fields-of-view to lower-throughput high-resolution elemental painting. Here we focused on qualitative measurement of chemically fixed targets, using protocols we – and others - typically use for EM examination of human tissues for diagnosis. Implementing other existing sample preparation protocols refined for research, like high pressure freezing followed by freeze substitution, may help to use EDX imaging in quantitative analysis and also allow determination of certain ions at high resolution in biosamples. Broad application of EM-EDX will allow experimental analysis on large-scale tissues using endogenous elements, multiple stains, and multiple markers and thus brings nanometer-scale ‘color-EM’ as a tool to unravel molecular (mis)regulation in biomedicine.

## Methods

### Tissue and sample preparation

Embedded tissue blocs in EPON (Serva) were used as described before^15^. Alternatively, in the case of osmium-free EPON embedding, tissue was post-fixed with 1% tannic acid (BDH chemicals, UK) in 0.05 M maleate buffer pH 5.15 for 40 minutes at room temperature. Furthermore, for additional membrane fixation, tissue was incubated in 1% poly-phenylenediamine (PPD, Merck-Millipore, Germany) in 70% ethanol for 20 minutes as one of steps during dehydration via graded ethanol series. All methods were carried out in accordance with relevant guidelines and regulations and the university ethical board for animal studies from the University Medical Center Groningen (UMCG), The Netherlands approved all animal experiments reported in this study^15^.Ultrathin sections (100 nm) were cut and collected on formvar single slot pyrolytic carbon grids (EMS, Hatfield, Pennsylvania).

### Post-embedding immuno-labeling

Grids were incubated with tissue facing down on droplets on parafilm at RT. First, samples were etched with 1% periodic acid (Merck, New Jersey) in milliQ water for 10 minutes to increase antigenicity, and rinsed in milliQ (4 × 5 min). This was followed by 30 minutes blocking with 1% bovine serum albumin (BSA; Sanquin, The Netherlands) in tris-buffered saline (TBS), pH 7.4. For insulin and amylase labeling an alternative blocking buffer (1% BSA-c (Aurion, Netherlands), 1% cold water fish skin gelatin (Sigma-Aldrich), 35 mM lysine (Fluka), and 1% normal goat serum (Jackson Immunoresearch, UK) in TBS, pH 7.4) was used for both blocking and antibody dilutions. Next, anti-insulin (guinea pig; 1:1000 in 1% BSA/TBS, Invitrogen) combined with either monocolonal 1H6 (1 μg/ml in 1% BSA/TBS)^19^ or anti-amylase (rabbit; 1:50 in blocking buffer, Sigma-Aldrich), or anti-glucagon (rabbit; 1:50 in 1% BSA/TBS, Thermo Fisher) alone incubated for 2 hours, rinsed in TBS (4 × 5 min), followed by incubation for 1 hour with biotinylated secondary antibody (donkey-anti-guinea pig; 1:400 in 1% BSA/TBS, Jackson Immunoresearch) and subsequent rinsed in TBS (4 × 5 min). Finally, streptavidin-conjugated QD655 (1:1000 in 1% BSA/TBS; Life technologies, California) and secondary goat-anti-mouse antibody for 1H6 or goat-anti-rabbit for amylase conjugated to 10 nm gold (1:100 in 1% BSA/TBS; BBI solutions, United Kingdom), or goat-anti-rabbit-conjugated QD655 (1:1000 in 1% BSA/TBS; Life technologies) for glucagon were added for 1 hour and subsequently rinsed in TBS (4 × 5 min) and 0.1 M sodium cacodylate (2 × 5 min).

### Microscopy

#### Large-scale STEM (nanotomy)

A large area scan using Scanning Transmission Detection (STEM) was made using a Zeiss supra55 SEM with Atlas as described before^18^. From this dataset smaller areas covering several cell types were selected for EDX analysis. Pre-exposure to stabilize samples was carried out at low magnification, depending on sample area. Typically, for the complete islets, 80 kV was used for 1 hour in a TEM. We note that after careful pre-exposure, we analyzed samples without signs of electron-beam induced damage or carbon deposition visible when comparing SEM images obtained before and after inspection, even after EDX maps acquisition times exceeding 1,5 hours, i.e., pixel exposure times >1 ms (excluding time for drift correction procedures), and after spectral point acquisition times of ~30 s.

#### EDX-analysis

The experiments were performed on a FEI Verios SEM equipped with an Oxford Instruments X-Max 80 mm^2^ Solid State EDX detector. As stated by the supplier, when the detector is fully inserted, the distance between the sample and the EDX window is 35.81 mm for a working distance of 5 mm and a tilt angle of 35°. Hence, the collection angle is 0.061 sr. For practical reasons the position might not be identical, since we optimized the position for collecting the maximum number of X-ray counts at 4 mm working distance. It is difficult to measure the exact distance between the window and the sample. The EDX detector has supplier-stated energy resolution of ≤ 127 eV at the Mn Kα line, ≤64 eV at the F Kα line, and ≤56 eV at C Kα, all at count rates of 20,000 cps. Peak position and resolution on the Mn Kα do not change more than 1 eV between 1,000 and 100,000 cps as calibrated upon installation. Au nanoparticles with a mean diameter of 2.7 nm dispersed on a thin Carbon support grid were detected in EDX, indicating that for high-density probes spatial resolution in EDX can approach the SEM probe size.

The imaging of the region of interest is performed using the ICD (In Column Detector) available in the Verios SEM at a resolution of 3072 × 2207 pixels, with dwell time of 30 us per pixel. All the regions of interest were imaged at 15 kV acceleration voltage and 26 nA current, at 4 mm working distance, in Ultra High Resolution (UHR) mode. The EDX maps were all taken at 15 kV primary beam energy and 26 nA current, at a working distance of 4 mm, in UHR mode. The Oxford Instrument AZtec software was used for the acquisition of EDX maps and point & ID analysis. The resolution of the maps is 2048*1408 pixels, with dwell time 20 us per pixel. The process time, i.e. the time over which the voltage signal generated by the detector is averaged, was set to 4 (on a scale from 1 to 6) in order to optimize the acquisition rate and to reduce the voltage noise, and we selected 2048 channels, that gives an energy window of 10 eV per channel. In case of point & ID analysis, 4096 channels were used, with an energy of 5 eV per channel. Because of the long acquisition time for the EDX maps, sample and stage drift plays an important role. For that reason, the AZtech software drift correction option was activated, meaning that after a certain amount of time, determined by the software based on the drift speed, an EM image is acquired, which is compared to an original image taken at the start of the EDX map. The software automatically detects particles or shapes in the image, from which it calculates the drift and compensates for it. The life-time, i.e. the effective time in which the spectra is acquired and integrated, and the collected number of frames for each field of view are listed in Supplementary Table S1.

### Image processing

In Fiji (http://fiji.sc/), three selected elements were merged as RGB. All image processing has been applied to the whole image in a linear fashion using Fiji and/or Adobe Photoshop. To discriminate 10 nm immunogold and QD655 nanoparticles upon immunolabeling of amylase and insulin, semi-automated annotation using the Fiji macro “golddigger” was performed^35^.

## Additional Information

**How to cite this article:** Scotuzzi, M. *et al*. Multi-color electron microscopy by element-guided identification of cells, organelles and molecules. *Sci. Rep.* **7**, 45970; doi: 10.1038/srep45970 (2017).

**Publisher's note:** Springer Nature remains neutral with regard to jurisdictional claims in published maps and institutional affiliations.

## Supplementary Material

Supplementary Information
