# Supplementary material for: Multi-color electron microscopy by element-guided identification of cells, organelles and molecules
Source: Sci Rep. 2017 Apr 7;7:45970. doi: 10.1038/srep45970 (PMC5384080; doi:10.1038/srep45970)
Supplement: Supplementary Information [file srep45970-s1.doc]

***Supplementary Materials***

**Multi-color electron microscopy by element-guided identification of cells, organelles and molecules**

Marijke Scotuzzi ^1,3^, Jeroen Kuipers ^2,3^, Dasha I. Wensveen ^1,3^ , Pascal de Boer ^2^, Kees (C.) W. Hagen ^1^, Jacob P. Hoogenboom ^1,4*^ & Ben N. G. Giepmans ^2,4*^

^1^Dept. Imaging Physics, Delft University of Technology, Delft, The Netherlands. ^2^Dept. Cell Biology, University Medical Centre Groningen, University of Groningen, Groningen, The Netherlands. ^3,4^denotes equal contribution. **correspondence to: Jacob P. Hoogenboom* [*j.p.hoogenboom@tudelft.nl*](mailto:j.p.hoogenboom@tudelft.nl) *or Ben N. G. Giepmans* [*b.n.g.giepmans@umcg.nl*](mailto:b.n.g.giepmans@umcg.nl)

**Abstract:** Cellular complexity is unraveled at nanometer resolution using electron microscopy (EM), but interpretation of macromolecular functionality is hampered by the difficulty in interpreting grey-scale images and the unidentified molecular content. We perform large-scale EM on mammalian tissue complemented with energy-dispersive X-ray analysis (EDX) to allow EM-data analysis based on elemental composition. Endogenous elements, labels (gold and cadmium-based nanoparticles) as well as stains are analyzed at ultrastructural resolution. This provides a wide palette of colors to paint the traditional grey-scale EM images for composition-based interpretation. Our proof-of-principle application of EM-EDX reveals that endocrine and exocrine vesicles exist in single cells in Islets of Langerhans. This highlights how elemental mapping reveals unbiased biomedical relevant information. Broad application of EM-EDX will further allow experimental analysis on large-scale tissue using endogenous elements, multiple stains, and multiple markers and thus brings nanometer-scale ‘color-EM’ as a promising tool to unravel molecular (de)regulation in biomedicine.

***Supplementary Materials***:

Materials and methods

*Figure S1.* Additional element-specific images as indicated. See Fig. 1 for details.

*Figure S2.* Additional element-specific images as indicated. See Fig. 2 for details.

*Figure S3.* Additional element-specific images as indicated. See Fig. 3 for details.

*Figure S4.* EDX spectra comparison for different granules.

*Figure S5.* Glucagon granules in Osmium-free tissue with quantum dot labeling.

*Figure S6.* EDX spectra on Au or quantum dot-labeled areas in Fig. 2

*Table S1.* EDX acquisition characteristics per FOV

*External databases:* Full resolution STEM data of Fig. 1a, 2a, 3a, 4 is available via full resolution large-scale EM data is available via [www.nanotomy.org](http://www.nanotomy.org/)

***
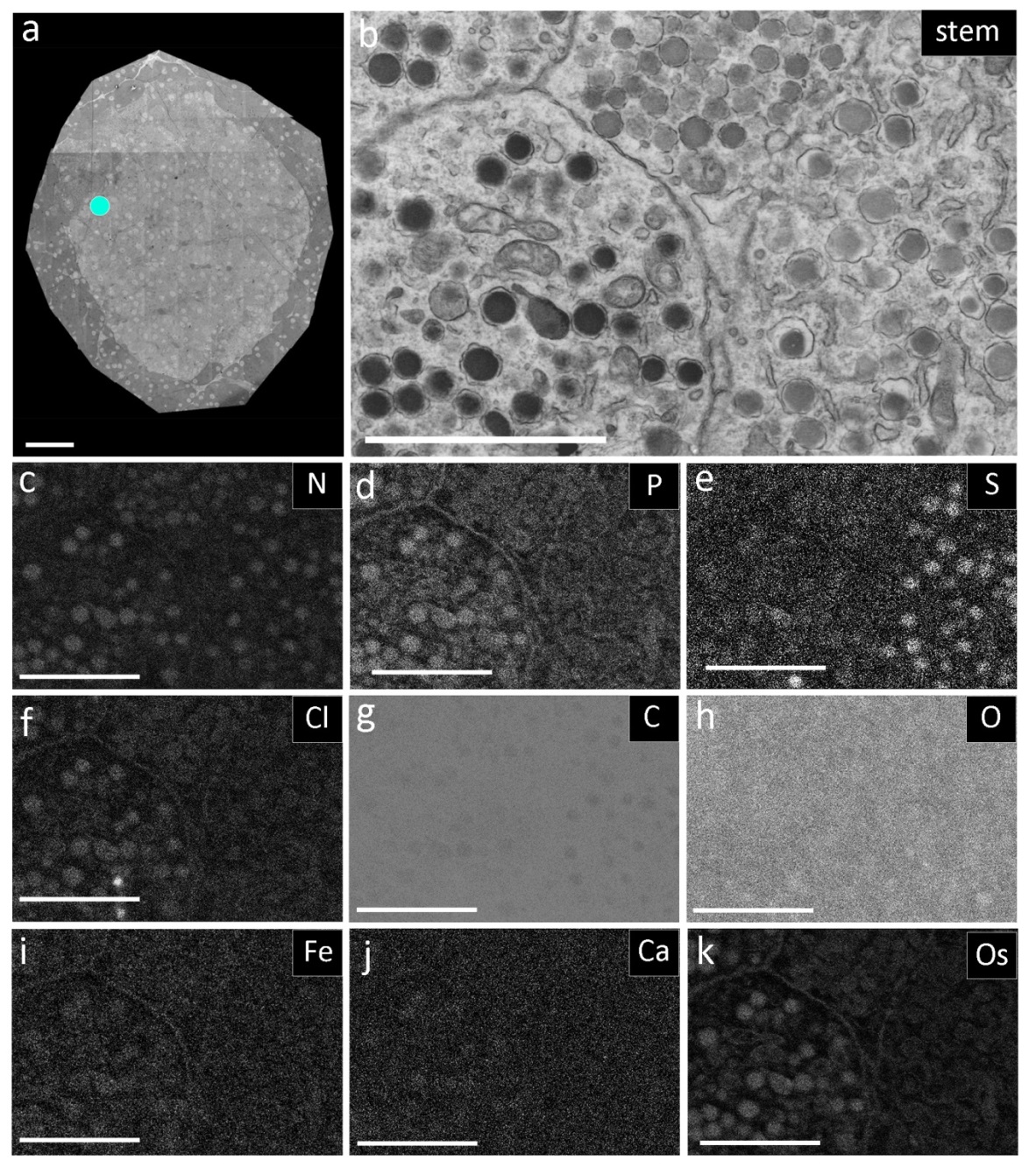
***

**Figure S1.** Additional element-specific images as indicated. See Fig. 1 for details.


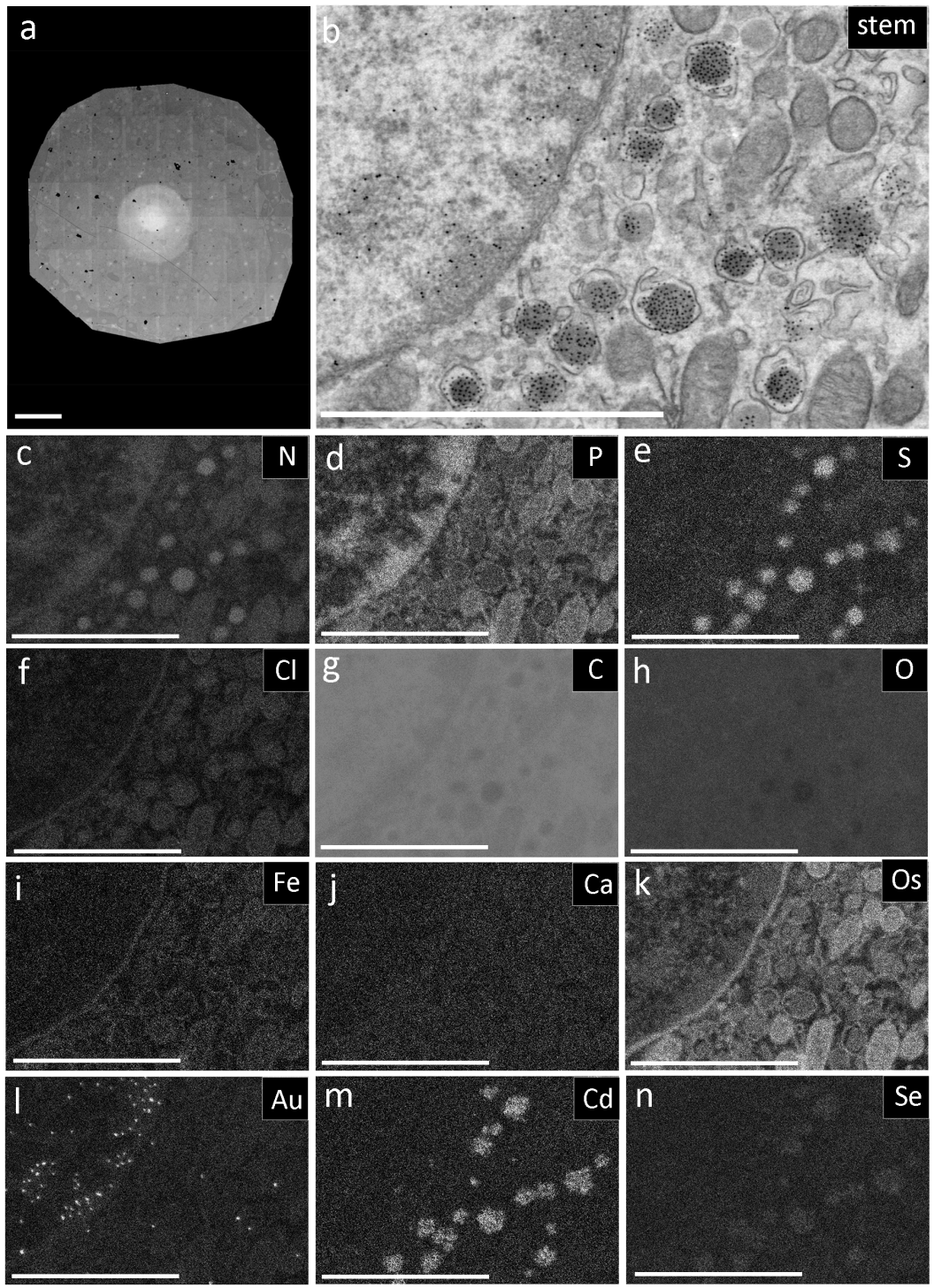


**Figure S2.** Additional element-specific images as indicated. See Fig. 2 for details.


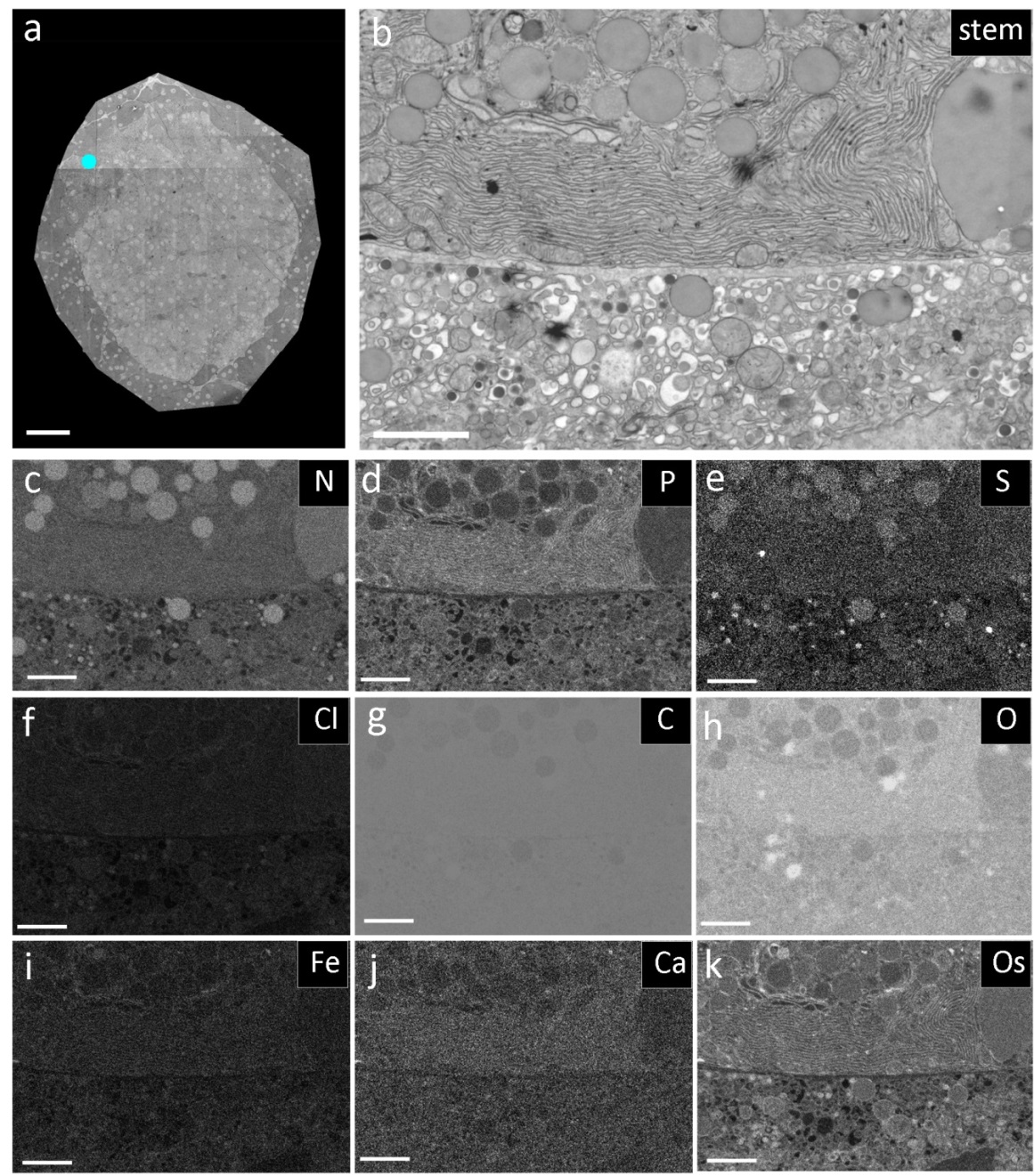


**Figure S3.** Additional element-specific images as indicated. See Fig. 3 for details.


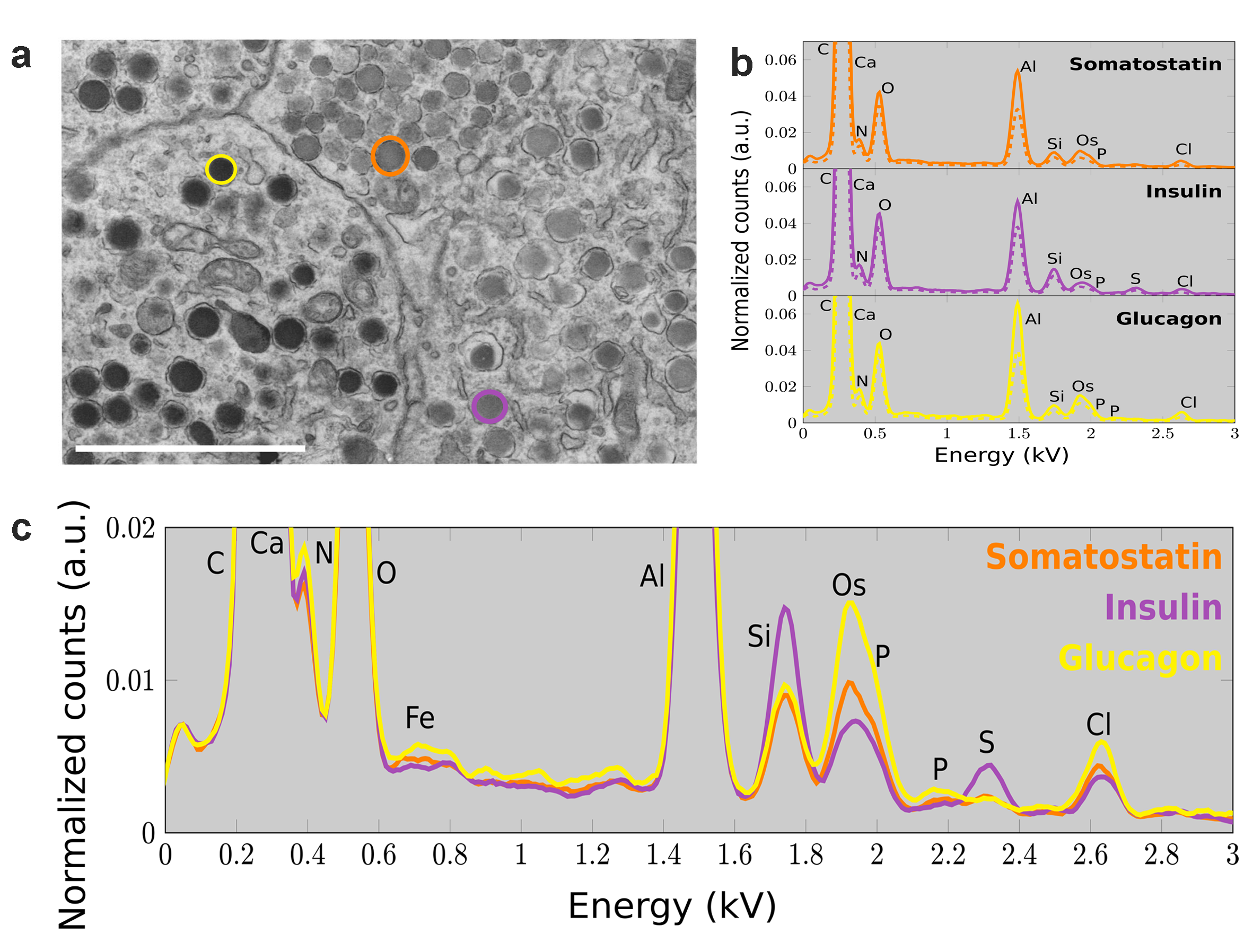


**Figure S4.** EDX spectral differences between different hormone-containing granules. (**a**) Same area as in manuscript Figure 1 with circles indicating the granules for which EDX spectra are shown in (b). (**b**) EDX spectra for the respective granules in (a). Drawn lines denote spectra obtained by averaging the data collected in the spatial maps over the indicated granules. Dashed lines indicate spectra obtained by 30 second integration on a single spot within the granule. All spectra are normalized to the C peak and smoothed using a standard Matlab routine. All spectral features in the spectra obtained from the EDX maps are qualitatively reproduced in the spot integration curves, confirming the validity of the acquisition time for the EDX maps. The pronounced Al signal comes from sampleholder, probably from back-scattered electrons generated in the sample that hit the sample holder. (**c**) Comparison of EDX spectra obtained for the three different granules, revealing the increased level of S in insulin granules, P in glucagon granules, and relative absence of both for somatostatin. Note also the pronounced differences in Os staining level corresponding to the granule interpretation in standard grey-scale EM. Scalebar in (a) 2μm. Experimental settings: 15kV beam energy at 26nA, EDX: process time 4, 4096 channels, 5eV per channel.


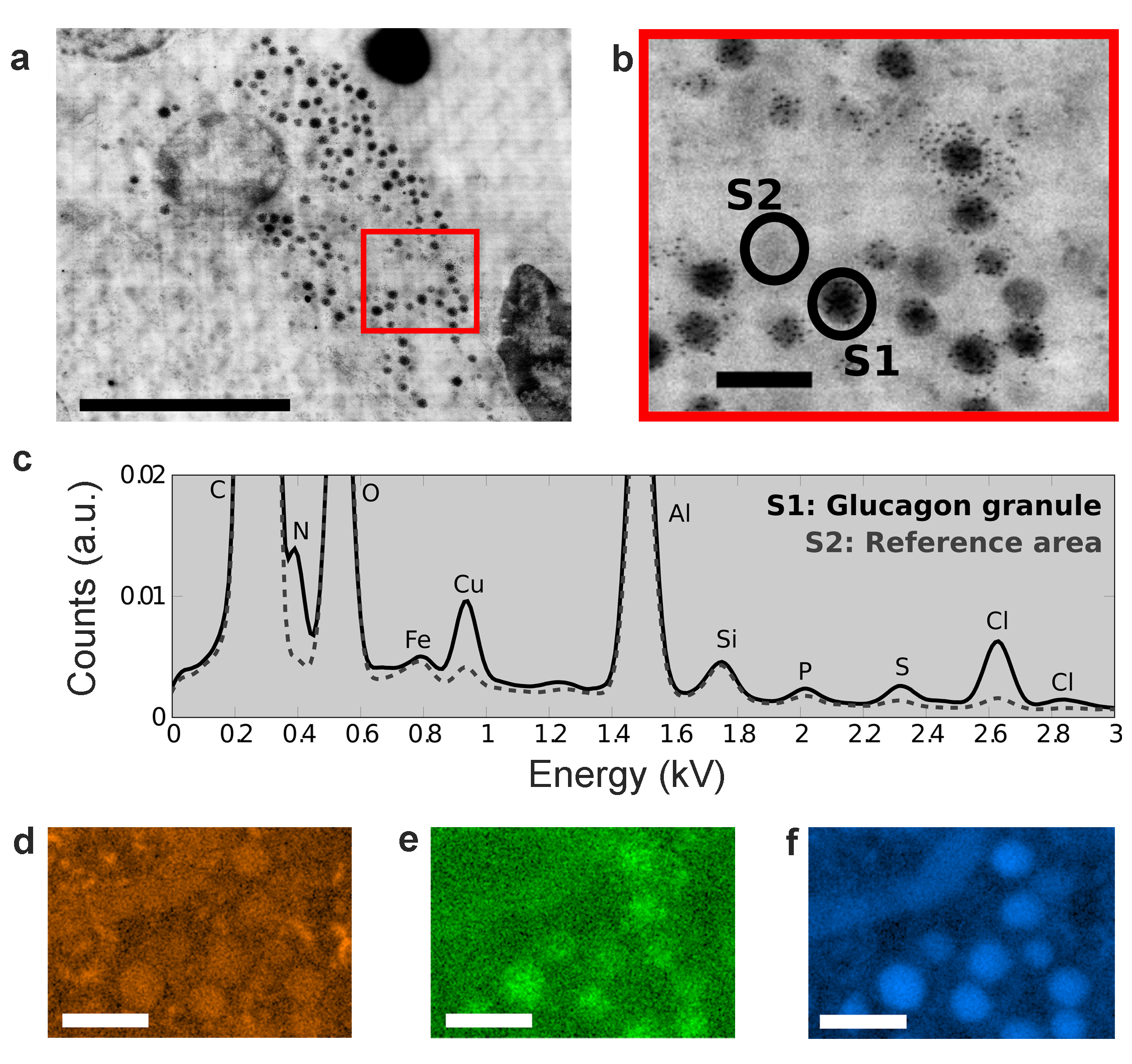
**Figure S5.** EDX data on glucagon-producing alpha cell using an Osmium-free sample preparation. Quantum dots are labelling glucagon to allow identification of the glucagon granules (**a**) SEM image of an alpha cell. (**b**) Magnified image of the boxed area in (a) with clear visibility of QDs on the granules. (**c**) EDX spectra obtained by integrating the spectral maps over the glucagon granule and the reference area indicated in (b). (**d, e, f**) EDX maps of the area in (b) depicting the signal for the indicated spectral lines for (d) P, (e) S, and (f) N. Note how the (speckled) S signal follows the distribution of the ZnS-shelled quantum dots, visible especially in the upper right corner. The glucagon granules are clearly discerned in the P map. Sample preparation was similar to that for the data in the manuscript, except for the omission of Osmium and a section thickness of 300 nm. The spectra in (c) have been normalized to the C peak and smoothed using Matlab. Scale bars are 5 µm in (a) and 500 nm in the other panels.


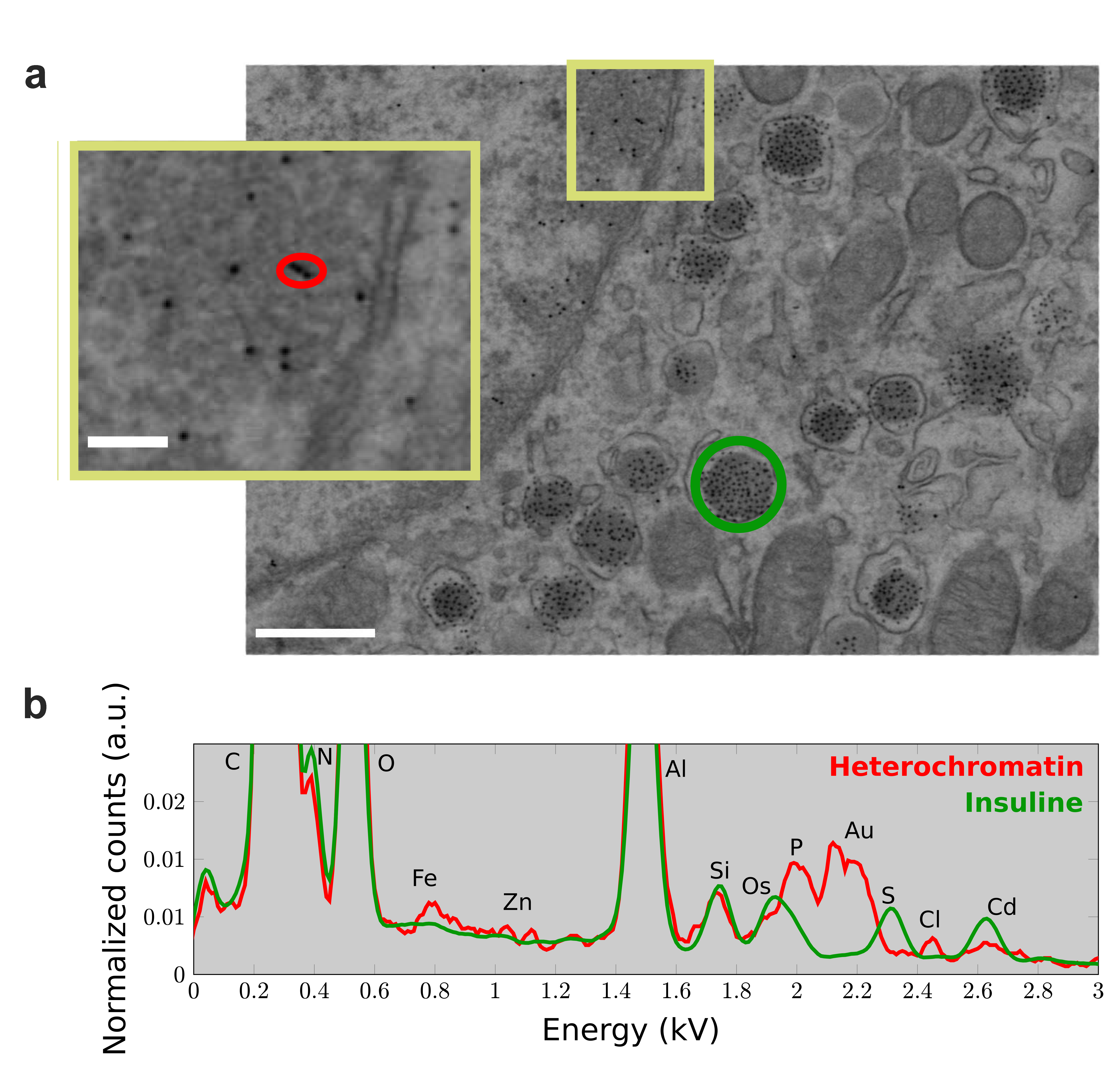


**Figure S6.** EDX spectra comparison for quantum dot and gold nanoparticle labelled sample. (Manuscript Fig. 2). (**a**) Same area as in Fig 2b with circles indicating regions over which EDX spectra were averaged for (b). Inset is shown for the area indicated with yellow rectangle. (**b**) EDX spectra averaged over the indicated areas. Au signal (targeted to 1H6) is clearly visible in conjunction with a strong P peak from heterochromatin. In the green area, Cd is clearly present originating from the quantum dots core, together with weaker Zn from the quantum dot shell. Note that the S peak appears slightly more pronounced compared to, e.g., Os and Cl than in Fig. 1b, presumably due to the additional presence of the ZnS quantum dots shell. Experimental settings as stated in the manuscript, spectra normalized to the C peak and smoothed using Matlab. Scale bar 500nm, and 100nm in the inset.

**Table S1**. EDX acquisition characteristics per FOV

| Data | Lifetime (10^3^ s) | Frames (n) |
| --- | --- | --- |
| Fig.1 | 1.5 | 25 |
| Fig.2 | 3.7 | 64 |
| Fig.3 | 5.8 | 100 |
| Fig.4 | 5.8 | 100 |
